# Levels and trends of adolescent marriage and maternity in West and Central Africa, 1986-2017

**DOI:** 10.7189/jogh.11.13001

**Published:** 2021-08-10

**Authors:** Vera Sagalova, Jonathan Garcia, Till Bärnighausen, John Ntambi, Roger Sodjinou, Noel Marie Zagre, Sebastian Vollmer

**Affiliations:** 1 Heidelberg Institute of Global Health, University of Heidelberg, Germany; 2 Department of Economics and Centre for Modern Indian Studies, University of Goettingen, Germany; 3 United Nations Children’s Fund (UNICEF), West and Central Africa Regional Office, Dakar, Senegal; 4 UNICEF Area Representative for Gabon and São Tomé and Príncipe and to the ECCAS, Libreville, Gabon; 5 United Nations Children’s Fund (UNICEF), Niamey, Niger

## Abstract

**Background:**

The world has made considerable progress in the reduction of adolescent maternity and early marriage. However, this progress has been uneven, with many countries finding themselves far from achieving the Sustainable Development Goals in this dimension. We assessed levels and trends over time in adolescent marriage and maternity prevalence within the West and Central African region as well as their correlation with select macro-level indicators for income and social institutions.

**Methods:**

We estimated country-specific prevalence rates using survey data (pooled cross-sectional) conducted between 1986 and 2017. The pooled sample provides information on 262 721 adolescent girls between the ages of 15 and 19. We assessed the relative country-level trends by comparing prevalence rates from the first and latest available survey in each country. We further analyzed regional trends by country income group (low- and middle-income) and examined the association of prevalence rates with measurements of gender discrimination and social institutions at the country-level. Estimations were conducted using survey weights and country-specific weights for population shares in the pooled sample.

**Results:**

Prevalence of adolescent maternity declined from 30.1 percent (95% confidence interval (CI) = 29.6%-32.2%) in the 1990s, to 28.7 percent (95% CI = 27.9%-29.6%) in the 2000s and 26.2 percent (95% CI = 25.4%-27.1%) in the 2010s. Adolescent marriage rates decreased from 37.3 percent (95% CI = 35.5%-39.1%) in the 1990s to 27.5 percent (95% CI = 26.5%-28.6%) in the 2000s, and to 24.9 percent (95% CI = 24.1%-25.7%) in the 2010s. Between 1986 and 2017, adolescent marriage decreased in all countries except for the Central African Republic (with a rise from 39% to 55%) and Niger (56% to 61%). The prevalence of adolescent maternity decreased in all but three countries: Congo, Dem. Rep. (25% to 37%), Niger (36% to 40%), and the Central African Republic (36% to 49%). When grouped by income level, the prevalence was 8 percentage points higher in low-income countries than in middle-income countries in both outcomes. We did not establish any statisticly significant association between adolescent marriage and maternity with country-level measures of discrimination against women. However, we found evidence of an association between specific legal measures of protection against early marriage and lower prevalence rates for both early marriage and maternity.

**Conclusions:**

Despite considerable progress in the reduction of adolescent maternity and marriage over the last 30 years, current levels of both indicators remain overall high in the WCA region, with high heterogeneity across individual countries. Countries with higher income level and higher standard in legal protection of young girls perform consistently better on both indicators. The prevalence rates of adolescent marriage and maternity reversed over the course of three decades, so that nowadays adolescent maternity rates exceed adolescent marriage rates in most countries. Further research is needed to understand the weak or non-existent association between adolescent marriage and maternity with gender discrimination and social institutions.

Adolescent maternity and marriage continue to be a major public health concern, especially for low- and middle-income countries, due to their implications for girls’ sexual and reproductive health, as well as their lasting consequences for girls’ educational attainment, income-earning potential, and other aspects of human development [1-3]. Early marriage and childbirth go hand in hand: it is estimated that around 95 percent of the world’s births to adolescent mothers occur in developing countries and that 90 percent of those births happen within a marriage or a union [4]. In developing countries, around 16 million older adolescents (girls aged 15 to 19 years) give birth every year [4], and approximately 650 million girls and women alive today were married before their 18th birthday [5].

Despite considerable progress in the reduction of adolescent maternity and early marriage, a large number of countries are not on track to achieve the global goal of reducing and subsequently eradicating early marriage and maternity [6]. Given the current pace of progress, the global proportion of women married as adolescents is projected to be as high as 18 percent by 2050 [7].

Furthermore, the so far uneven progress in reaching these targets across regions and countries within these regions, as well as opposing trends in population growth across countries, have widened global disparities in adolescent maternity- and marriage-rates [4,8].

In West and Central Africa (WCA, UNICEF geographic definition), the region with the highest prevalence rates of adolescent pregnancy and marriage, progress has been among the slowest in the world [9,10]. Fenn and co-authors estimate adolescent pregnancy to reach 34% in WCA [11], while adolescent marriage is estimated at 44% in the region [5]. Along with a rapidly growing population of girls (2.7% population growth and a fertility rate of 5.2 children per woman [10]), this has resulted in persistently high rates and an absolute increase in the number of adolescent wives and mothers globally.

Worldwide, decline in prevalence of adolescent maternity has been associated with national income, income inequality, and expenditures in education on macro level [12]. Evidence available as meta-analysis on various African countries [13,14] and on three countries from East Africa [15] suggests that wealth, urbanization, education, and religion are negatively associated with adolescent marriage and maternity rates. Likewise, empirical evidence suggests that, for the case of Africa, the countries with the highest rates of early marriage are also the countries with the highest rates of poverty and population growth [16]. In Sub-Saharan Africa, legislative efforts have significantly contributed to the reduction of early marriage, yet high levels persist [17,18]. Furthermore, despite being strongly linked, both phenomena are barely studied simultaneously and even less so for West and Central Africa. Trends and determinants of marriage and childbearing in three West African countries – Nigeria, Burkina Faso, and Niger – are investigated in a study by Avogo and Somefun [19], while UNICEF (2015) analyzes patterns and trends of pregnancy and family formation for 15 countries in WCA, confirming that they are strongly correlated and that both prevalence rates remain high.

The increase in data availability enabled through national representative surveys that capture social, health, and demographic information of households and their members, such as Demographic and Health Surveys (DHS) and Multi Indicator Cluster Surveys (MICS), facilitates the study of historical patterns of adolescent fertility and family formation [20]. This paper describes the levels and trends in adolescent marriage and maternity prevalence in WCA over the past 30 years. Furthermore, we assess the role of select protective factors – economic development, legal protection, and different measures of female empowerment – towards adolescent marriage and maternity by analyzing the correlation of those factors with our outcomes of interest at regional and country level, thus further closing the knowledge gap on adolescent marriage and maternity in WCA and providing additional context to policymakers within the region.

## METHODS

### Data

We pooled all Demographic and Health Surveys (DHS) and Multi Indicator Cluster Surveys (MICS) for 23 West and Central African countries that were conducted between 1986 and 2017 (a detailed list of countries and survey years for each outcome is provided in Table S1 in the **Online Supplementary Document**). The pooled sample provides data on 262 721 adolescent girls between the ages of 15 and 19. While there are some few younger adolescents in the pooled sample (n = 877), they were excluded from the present analyses as all of those observations stem from only two MICS surveys (Equatorial Guinea and Guinea Bissau 2000) and are generally not part of the MICS or DHS sampling design.

Moreover, we constructed and merged additional country-level variables based on the Social Institutions and Gender Index (SIGI) as well as World Bank income classification.

### Outcomes

Outcome variables are indicator variables for marriage and maternity. The marriage variable is coded as one if the adolescent girl was ever married. The maternity variable is coded as one if she has ever given birth or is currently pregnant. A weighted average of these binary variables aggregated on any geographical entity can hence be interpreted as prevalence in this entity.

### Exposure

Exposure variables include indicator variables for low-income and middle-income country according to the World Bank income group classification. A further exposure is the Social Institutions and Gender Index (SIGI) of the Organisation for Economic Co-operation and Developmen (OECD) and its select subindices. The SIGI is a composite index that measures discrimination against women in the civil liberties, physical integrity, access to resources, and discriminatory family code dimensions. Each dimension is a composite index of various indicators which are described in detail elsewhere [21].

In some instances where variation in some SIGI sub-indices within the West and Central African region was insufficient for a meaningful statistical analysis, we collapsed the indicator into fewer categories (eg, when the initial indicator is measured in five steps representing different values of legal protection we created broader categories with three steps – such as “fully protected”, “weakly protected”, and “not protected”).

The SIGI is constructed with comparable data across countries and its sub-indices range from 0 for no discrimination to 1 for absolute discrimination [21].

### Statistical analysis

Descriptive statistics were calculated using survey weights for individual surveys and in the pooled sample each country was additionally weighted with its population share. All regressions were simple linear models. Statistical analyses as well as sample generation have been performed using STATA 14 statistical package (StataCorp, College Station, TX, USA).

## RESULTS

**Figure 1:**
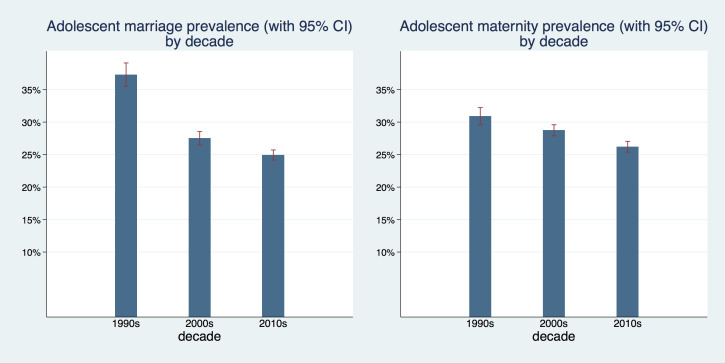
Prevalence of adolescent marriage and maternity 1990s, 2000s and 2010s.

**Figure 1** depicts the prevalence of adolescent marriage and maternity by decade (Table S1 in the **Online Supplementary Document** shows all countries that were included in the analysis with respective sample sizes by DHS survey phase). We observed a moderate decline in adolescent maternity from 30.1 percent (95% confidence interval (CI) = 29.6%-32.2%) in the 1990s, to 28.7 percent (95% CI = 27.9%-29.6%) in the 2000s and 26.2 percent (95% CI = 25.4%-27%) in the 2010s. The decline of adolescent marriage was more pronounced starting from 37.3 percent (95% CI = 35.5%-39.1%) in the 1990s, decreasing to 27.5 percent (95% CI = 26.5%-28.6%) in the 2000s, and to 24.9 percent (95% CI = 24.1%-25.7%) in the 2010s.

**Figure 2:**
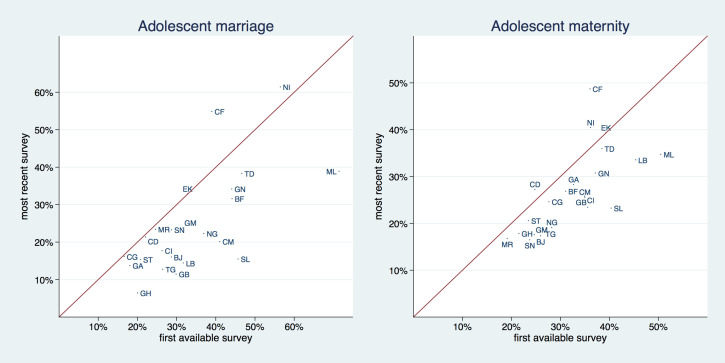
Trends over time – change in adolescent marriage and maternity prevalence by country (first and last survey). EK (Equatorial Guinea has only one survey available, hence it is situated exactly at the 45° line).

**Figure 2** and **Table 1** and **Table 2** allow for a graphical and analytical examination of trends in the prevalence of adolescent marriage and maternity on country level by comparing the first and last available survey. We provide the results (*P* values) of hypothesis tests (*t* test) in the last column of both tables. The solid line in **Figure 2** is a 45° line, the x-axis shows the prevalence for the first available and the y-axis for the most recent survey. Countries that are situated above the 45° degree line increased their respective prevalence and countries that are situated below the 45° degree line decreased it. The distance from the 45° degree line indicates the magnitude of the change.

**Table 1:** Adolescent marriage prevalence by country

	First available survey				Most recent available survey				
**Country**	**Year**	**Prevalence**	**95% CI**	**N**	**Year**	**Prevalence**	**95% CI**	**N**	***P*-value**
Benin	1996	0.29	(0.25-0.33)	1049	2014	0.16	(0.14-0.18)	3151	* <* 0.001
Burkina Faso	1992	0.44	(0.40-0.48)	1480	2010	0.32	(0.29-0.34)	3326	* <* 0.001
Cameroon	1991	0.41	(0.36-0.46)	949	2014	0.20	(0.18-0.23)	2101	* <* 0.001
Central African Republic	1994	0.39	(0.36-0.42)	1289	2010	0.55	(0.52-0.58)	2349	* <* 0.001
Chad	1996	0.47	(0.43-0.50)	1716	2014	0.38	(0.36-0.41)	3889	* <* 0.001
Congo	2005	0.17	(0.14-0.19)	1497	2014	0.16	(0.14-0.18)	2021	0.784
Congo, Dem. Rep.	2001	0.22	(0.20-0.24)	2933	2013	0.21	(0.19-0.24)	3981	0.618
Cote d'Ivoire	1994	0.26	(0.23-0.29)	1993	2016	0.18	(0.15-0.20)	2260	* <* 0.001
Gabon	2000	0.18	(0.16-0.21)	1613	2012	0.14	(0.11-0.17)	1806	0.030
Gambia	2000	0.32	(0.27-0.37)	1175	2013	0.24	(0.21-0.27)	2463	0.004
Ghana	1993	0.20	(0.17-0.23)	803	2014	0.06	(0.05-0.08)	1756	* <* 0.001
Guinea	1999	0.44	(0.41-0.48)	1332	2016	0.34	(0.32-0.37)	1902	* <* 0.001
Guinea Bissau	2000	0.30	(0.25-0.34)	1559	2014	0.11	(0.10-0.13)	2278	* <* 0.001
Liberia	1986	0.32	(0.27-0.36)	1169	2013	0.14	(0.12-0.17)	1915	* <* 0.001
Mali	1987	0.71	(0.66-0.77)	556	2015	0.39	(0.36-0.42)	3827	* <* 0.001
Mauritania	2007	0.25	(0.23-0.27)	2696	2015	0.23	(0.21-0.26)	2962	0.425
Niger	1992	0.56	(0.52-0.60)	1427	2012	0.61	(0.58-0.65)	1828	0.064
Nigeria	1990	0.37	(0.32-0.43)	1678	2016	0.22	(0.21-0.24)	6780	< 0.001
São Tomé and Príncipe	2008	0.21	(0.16-0.25)	518	2014	0.15	(0.12-0.19)	688	0.070
Senegal	1986	0.29	(0.26-0.32)	1426	2017	0.23	(0.21-0.26)	3920	0.004
Sierra Leone	2000	0.46	(0.42-0.50)	977	2017	0.15	(0.14-0.17)	3903	< 0.001
Togo	1988	0.27	(0.22-0.31)	724	2013	0.13	(0.11-0.15)	1733	< 0.001

CI – confidence interval

**Table 2:** Adolescent maternity prevalence by country

	First available survey				Most recent available survey				
**Country**	**Year**	**Prevalence**	**95% CI**	**N**	**Year**	**Prevalence**	**95% CI**	**N**	***P*-value**
Benin	1996	0.26	(0.22-0.30)	1049	2014	0.17	(0.15-0.19)	3151	< 0.001
Burkina Faso	1992	0.31	(0.27-0.35)	1480	2014	0.27	(0.23-0.30)	1714	0.085
Cameroon	1991	0.35	(0.31-0.39)	949	2014	0.26	(0.23-0.28)	2103	< 0.001
Central African Republic	1994	0.36	(0.33-0.39)	1289	2010	0.49	(0.46-0.52)	2349	< 0.001
Chad	1996	0.38	(0.36-0.41)	716	2014	0.36	(0.34-0.38)	3889	0.157
Congo	2005	0.28	(0.25-0.31)	1497	2014	0.25	(0.22-0.27)	2021	0.121
Congo, Dem. Rep.	2001	0.25	(0.23-0.27)	2933	2013	0.27	(0.25-0.30)	3981	0.126
Cote d'Ivoire	1994	0.35	(0.32-0.38)	1993	2016	0.26	(0.23-0.28)	2260	< 0.001
Gabon	2000	0.33	(0.30-0.36)	1613	2012	0.28	(0.24-0.32)	1806	0.074
Gambia	2000	0.25	(0.21-0.29)	1175	2013	0.18	(0.15-0.20)	2463	0.002
Ghana	1993	0.22	(0.19-0.24)	803	2016	0.18	(0.14-0.21)	964	0.108
Guinea	1999	0.37	(0.34-0.40)	1339	2016	0.31	(0.28-0.33)	1902	0.001
Guinea Bissau	2000	0.36	(0.32-0.39)	1559	2014	0.23	(0.21-0.26)	2278	< 0.001
Liberia	1986	0.45	(0.41-0.50)	1169	2016	0.34	(0.29-0.38)	895	< 0.001
Mali	1987	0.51	(0.45-0.56)	556	2015	0.35	(0.33-0.37)	5381	< 0.001
Mauritania	2007	0.19	(0.17-0.21)	2696	2015	0.17	(0.15-0.19)	2962	0.058
Niger	1992	0.36	(0.33-0.39)	1427	2012	0.40	(0.37-0.44)	1828	0.043
Nigeria	1990	0.28	(0.24-0.32)	1678	2016	0.19	(0.18-0.20)	6786	< 0.001
São Tomé and Príncipe	2008	0.24	(0.18-0.29)	518	2014	0.21	(0.17-0.24)	688	0.396
Senegal	1992	0.24	(0.21-0.26)	1426	2017	0.16	(0.15-0.18)	3920	< 0.001
Sierra Leone	2000	0.40	(0.37-0.44)	993	2017	0.23	(0.21-0.25)	3943	< 0.001
Togo	1988	0.27	(0.22-0.31)	724	2017	0.19	(0.15-0.22)	804	0.002

CI – confidence interval

The prevalence of adolescent marriage decreased in all countries with the exception of Central African Republic (from 39% to 55%) and Niger (56% to 61%). For a few countries – Congo Dem. Rep., Congo, and Mauritania – this reduction was negligible. Reduction in prevalence was largest (and statistically significant with *P* < 0.000) in Sierra Leone with a drop from 45% to 15% and Mali, where it declined from 71% to 39%.

The prevalence of adolescent maternity decreased in all but three countries: Congo, Dem. Rep. (25% to 37%), Niger (36% to 40%), and Central African Republic – where we observed both the highest increase and the highest current prevalence (36% to 49%) (with *P-*values of 0.121, 0.043, and * <* 0.001, respectively). Sierra Leone and Mali experienced the largest reduction in this indicator, as they already did with adolescent marriage – 40% to 23% and 51% to 35%, respectively *(P <* 0.001*)*.

**Figure 3:**
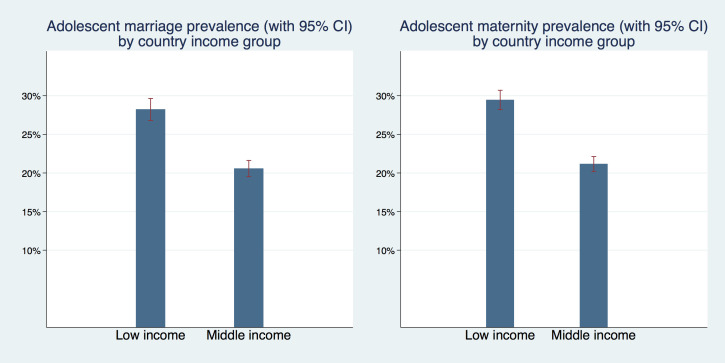
Adolescent marriage and maternity by country income group

**Figure 3** graphs the prevalence of adolescent marriage and maternity by country income level for a pooled sample of most recent surveys by country. Across both outcomes prevalence rates were higher in low-income than in middle-income countries. Adolescent marriage prevalence was 28.2% (95% CI = 26.8%-29.6%) in low-income countries and 20.6% (95% CI = 19.5%-21.6%) in middle-income countries. Adolescent maternity prevalence was 29.5% (95% CI = 28.2%-30.7%) in low-income countries and 21.1% (95% CI = 20.2%-22.1%) in middle-income countries.

**Figure 4:**
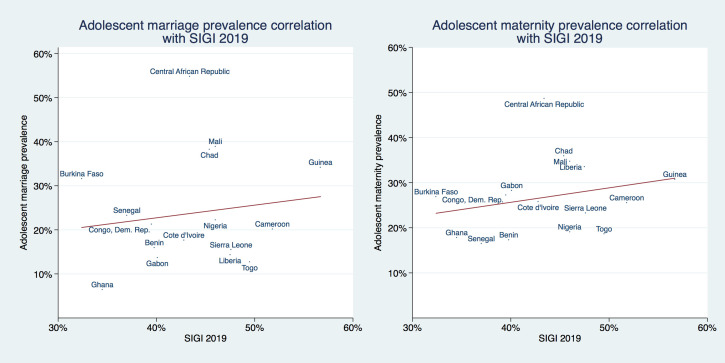
Association of adolescent marriage and maternity with SIGI 2019 index.

**Figure 4** plots the correlation of adolescent marriage and maternity prevalence with the combined SIGI score of each country. A fitted regression line suggests a weak positive correlation between adolescent marriage/maternity prevalence and the SIGI score, ie, countries with more discrimination against women had a higher prevalence for both outcomes. However, these positive correlations were not statistically significant (*P* = *.58* for adolescent marriage and *P* = *.28* for adolescent maternity). Please note that early marriage is one of the 21 subindices (one of the six variables included into the ‘discriminatory family code’ dimension) from which the SIGI is constructed, we elaborate on this within the discussion section.

Correlation between adolescent marriage and maternity prevalence with individual SIGI dimensions are shown in Figure S1 in the **Online Supplementary Document****.**

**Figure 5:**
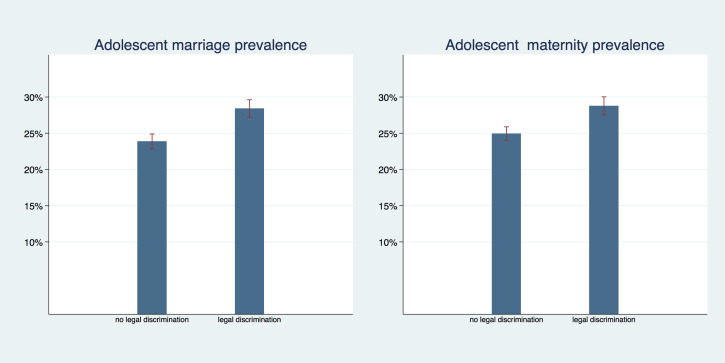
Adolescent marriage and maternity (with 95% CI) by legal discrimination of girls' marriage age.

Legal protection against early marriage, however, was associated with lower prevalence rates. In **Figure 5** we show the prevalence of adolescent marriage and maternity by level of legal discrimination against girls in child marriage laws; Countries without legal discrimination of girls (where the law guarantees the same minimum age of marriage to males and females) had an adolescent marriage prevalence of 24.9% (95% CI = 24.0%-25.9%) compared to 28.7% (95% CI = 27.5%-30.0%) in countries with legal discrimination against girls. The pattern is similar for adolescent maternity prevalence with 21.3% (95% CI = 18.6%-23.6%) in countries without legal discrimination against girls and 28.4% (95% CI = 27.2%-29.6%) in countries with legal discrimination against girls. In both outcomes there was no overlap in confidence intervals and *P* < 0.001.

## DISCUSSION

For most countries in West and Central Africa, we found that rates of adolescent marriage and maternity have decreased over time, which is in line with the trends experienced in the rest of the world [2,22-24] and other regions in Africa [13,18,25]. However, prevalence of both indicators is still high in the entire region. We estimated an average prevalence of 26.2% (95% CI = 25.4%-27.1%) for adolescent maternity and 24.9% (95% CI = 24.1%-25.7%) for adolescent marriage in the 2010s. In absolute values, this corresponds to roughly 5.9 million older adolescent girls who experienced early pregnancy and marriage in the region. High rates of adolescent marriage contribute to a vicious cycle of high fertility rates and rapid population growth [26], which exert additional pressure on economic opportunities and health of the population. Our results for both indicators are lower than those presented by UNICEF in their analysis of surveys for 15 countries in WCA between 2008 and 2014 [11], which estimates an average prevalence of child marriage of 43.8% and a rate of adolescent childbearing in the order of 34.3%. This difference in estimates could stem from the difference in countries sampled, the time frame, as well as weighting procedures.

Albeit the majority of countries experienced a reduction in prevalence rates of both indicators, this does not hold for Niger and the Central African Republic, which experienced an increase in the prevalence of adolescent marriage and maternity, as well as for the Democratic Republic of Congo with an increase in adolescent maternity rates. These countries are currently exposed to particularly high political and socio-economic volatility, in addition to recurrent emergencies such as high food prices, floods, and epidemics that increase children's vulnerability to violence, forced marriage, and involuntary migration [11]. Patterns identified for these countries call for additional research to facilitate a catching-up process tailored to these particular contexts.

Greater progress in the reduction of prevalence of adolescent marriage and maternity occurred in Mali and Sierra Leone, with a decrease in adolescent marriage prevalence rate of more than 30 percentage points and an adolescent maternity reduction of 16 and 17 percentage points, respectively, which almost halved the prevalence in Sierra Leone.

We do not attempt to explain what causes the different patterns in the trends across countries. However, we observed that for both variables, middle-income countries show lower prevalence rates than low-income countries. Likewise, we observed a decline of adolescent marriage and maternity with progressive economic development. These findings are in line with Santelli et al., who establish a negative association between adolescent birth rates and GDP levels [12].

While neither adolescent marriage nor maternity were correlated with general measures of discrimination against women, both were lower in countries that have higher levels of legal protection against early marriage of girls. Our results are coherent with findings by Maswikwa and co-authors that consistent minimum marriage age laws protect against the exploitation of girls [17].

This study is subject to several limitations: for one, we do not claim to identify any causal relationships, as the identification strategy in this cross-sectional pooled sample does not allow for such a claim. Moreover, the composition of sub-samples in our longitudinal analysis (marriage/maternity rates by decade) varies across decades: the sub-sample from the first decade, the 1990s, consists of only 13 countries, the subsequent decades, the 2000s and 2010s contain 23 and 22 countries, respectively. However, limiting the analysis to only the original 13 countries available in the 1990s as a robustness check (results not provided, available upon request) affects the estimates only marginally (<1 percentage point). Hence we feel safe to assume that composition effects are negligible in this context.

Furthermore, we provide graphic results of a regression of the outcome “adolescent marriage” on the SIGI composite indicator. However, “prevalence of early marriage” is one of 21 subindices that comprise the SIGI, and hence our model is slightly misspecified and the results of this regression have to be interpreted with caution, as we explain variation in the outcome, at least to a minor part, with its own variation, thus potentially overestimating the true coefficient. However, this does not undermine our conclusion of “no asscocation” between the SIGI and adolescent marriage in the region. Another potential drawback is the lack or unreliability of censuses in many sub-Saharan African countries which might affect our use of population weights negatively. Nevertheless we feel confident that population estimates provided by the UN Population Division which has developed a sophisticated framework that incorporates censuses, vital registration, official statistics, and household surveys as well as various additional sources from international and regional organizations, are sufficiently accurate and do not introduce any bias in our estimates. Household surveys have been subject to scrutiny and studied to assess their quality and consistency. Furthermore, Pullum [27] suggests that using weights could contribute to correcting bias which may result from ignoring the variation in sampling fractions used for the surveys.

An additional limitation of this study is that, while we generally speak of ‘adolescent’ maternity and marriage, we only investigate ‘older adolescent’ maternity and marriage, which are defined as those of adolescents aged 15-19. ‘Younger adolescents’ (aged 10-14), who constitute a smaller yet dramatically vulnerable group of mothers and brides, are systematically left out in the sampling strategy of large household surveys. Finally, while the use of country-level estimates might fail to depict within-country heterogeneities, the appropriate use of nationally representative surveys offer valuable information to understand the progress and needs at the regional level.

## CONCLUSION

We identified and quantified the progress in reduction of adolescent maternity and marriage prevalence within the West and Central Africa region as a whole and in region’s individual countries. Our results emphasize the considerable reduction within the last 30 years, especially in the adolescent marriage indicator, for the entire region on average and most of the countries included in the study individually. Simultaneously, reduction in adolescent maternity rates has, at least on average, been much more conservative and the corresponding rates in individual countries have declined less than marriage prevalences. This, while still a progress at the first glance, may actually leave young girls potentially more vulnerable: we found marriage rates to exceed maternity rates in the 1990s. However, this has reversed in the 2000s, and in 2010s, on aggregated level, 26.2% of young girls became mothers but only 24.9% of adolescent girls were married. This may reflect evolved preferences towards marriage and reduced stigma in out-of-wedlock births and would hence imply a positive development if young women actively choose not to marry even after becoming pregnant in some cases. It may, as well, signify an increasing vulnerability and disadvantage if it is a reflection of changed cultural norms but not changed preferences of adolescent mothers themselves. In the present paper, we do not perfom any analyses with this double outcome, however, exploring this issue is an interesting area for future research which, ideally, should be complemented by mixed-methods studies that investigate this interplay of personal preferences and cultural norms with regards to adolescent marriage and maternity.

We also stress that the current levels of adolescent maternity and marriage remain high and that additional factors such as rapid increase in the number of girls in the region represent a major risk to the achievement of the SDGs targets on adolescent maternity and marriage.

Similar to other studies of global and regional trends, our study found that a country's economic development is negatively associated with prevalence rates of maternity and marriage among adolescent girls. However, economic growth cannot be claimed to be causally linked to reduced adolescent maternity and marriage. It rather operates via complex pathways which may include expanded educational and economic opportunities for young girls, enhanced health service provision (and in particular reproductive health), and increased agency of young women overall. To explore these linkages and pathways, better, more consistent, and detailed data on various socio-economic and socio-cultural indicators would be necessary.

## Additional material


Online Supplementary Document
